# Localized delivery of coding nucleic acids into adherent cells by *in situ* electroporation: integrated impedance-based monitoring allows for *loss-of-function* or *gain-of-function* screening

**DOI:** 10.1038/s41598-026-63882-5

**Published:** 2026-07-29

**Authors:** Anne-Kathrin Grimm, Sonja Balk, Achim Göpferich, Miriam Breunig, Simone Aubele, Anne Zemella, Joachim Wegener

**Affiliations:** 1 Institut für Analytische Chemie, Chemo- und Biosensorik, Universität Regensburg, Regensburg, Germany; 2 Institut für Pharmazie, Universität Regensburg, Regensburg, Germany; 3 Institutsteil Bioanalytik und Bioprozesse, Fraunhofer-Institut für Zelltherapie und Immunologie, Potsdam, Germany; 4 Fraunhofer-Institut für Elektronische Mikrosysteme und Festkörper-Technologien EMFT, Regensburg, Germany

**Keywords:** In situ electroporation (ISE), Electric cell-substrate impedance sensing (ECIS), Intracellular delivery, Gain-of-function, Loss-of-function, Phenotypic assays, Biological techniques, Biotechnology, Cell biology

## Abstract

Coding nucleic acids (NAs) like DNA, mRNA or siRNA hold endless potential to engineer cells with respect to their genome and proteome. However, minimally invasive delivery of NAs across an intact plasma membrane into the cytosol of living cells remains a technical challenge. Electroporation creates transient pores in the plasma membrane by applying well-defined electric fields for fractions of a second allowing NA transfer across the membrane. *In situ electroporation* (*ISE*) applies the electric field to adherent cells growing on the surface of planar, thin-film electrodes deposited on the bottom of the culture well. This study reports on the localized delivery of plasmid DNA, mRNA, siRNA and aptamers into different mammalian cell lines by *ISE*. Fluorescence microscopy verified successful delivery of labeled NAs themselves (siRNA, aptamers) or expression of the proteins they encoded (DNA, mRNA). Impedance readings of the cell-covered electrodes before and after *ISE* allowed tracking the invasiveness of the pulse, the recovery of the cells within 30 min and the phenotypic impact of the NAs inside the cells. Loading the cells with siRNA specifically designed to knock down gene transcripts essential for survival, led to the onset of cell death within 20 h after the pulse. The combination of *ISE*, specific NAs and impedance-based cell monitoring paves the way for a new class of *gain-of-function* or *loss-of-function* experiments.

**Supplementary Information:**

The online version contains supplementary material available at 10.1038/s41598-026-63882-5.

## Introduction

Cells maintain and separate the chemical composition of their cytoplasm from the extracellular environment by means of their plasma membranes. Some molecules are able to pass through the membrane, either by simple diffusion due to their lipophilicity and small size, or with the help of transport proteins residing in the membrane. Other molecules cannot cross the plasma membrane and are excluded from the cytosol because of their hydrophilicity, charge and size. The majority of larger biomolecules enabling molecular recognition such as nucleic acids, proteins, peptides, enzymes and many drugs belong to this latter group of compounds. Their use is limited by their inability to reach target sites within cells despite of their great potential for specific intracellular manipulation or as diagnostic tools even in basic research. For this reason, various strategies and technologies have emerged enabling efficient, rapid and ideally non-invasive delivery of the aforementioned biomolecules into the cytosol. Getting them ‘in’ allows for highly specific modulations of cell physiology and cell functions. This is particularly true for different nucleic acid (NA) species like DNA, mRNA, siRNA or aptamers as the latter have often been optimized for targeted cell engineering or manipulation. They are in the focus of this study.

Many of the established intracellular delivery strategies for NAs rely on membrane-mediated uptake mechanisms (endocytosis), membrane destabilization/permeabilization or both. In *chemical transfection* of nucleic acids, cationic lipids (*transfection reagents)* are typically mixed with the NAs of interest (e.g., mRNA, siRNA or DNA) to form a complex (*lipoplex*) that facilitates adhesion to the cell membrane, followed by non-specific or receptor-mediated endocytosis. Using this pathway, endosomes or lysosomes are often the NAs first (and final) intracellular destination. For successful delivery into the cytosol as free molecules, an *endosomal escape* must occur but is often limiting^1^. It often remains vague whether cationic lipids or other transfection reagents can overcome the endosomal fate of NAs and whether cytotoxic effects are fully avoidable^2^.

*Physical transfection* techniques overcome the membrane diffusion barrier by physical means^3^. Electroporation, which is in the focus of this study, induces transient membrane permeabilization enabling the direct introduction of external molecules into the cytosol via pores or by means of membrane destabilization^4–6^. Numerous studies have demonstrated its effectiveness in delivering exogenous nucleic acids into the cytoplasm or even the nuclei of living cells^7–9^. Most in vitro studies using electroporation have been conducted with cells in suspension. However, there is increasing interest in applying electroporation also to adherent cells (*in situ electroporation*,* ISE*). In *ISE*, the cells are attached and spread on conducting surfaces that are used to apply the electric field. Since the cells experience the elevated electric field with their cytoskeleton, cell-cell and cell-matrix contacts being fully established, we found the membrane to reseal within less than one second after the electric field has been switched off while the complete morphological recovery of the cells takes minutes to hours^10^. Thus, *ISE* provides a minimally invasive means to manipulate even very sensitive and fragile cell types like primary cells or stem cells.

This study integrates *ISE* of cells, adherently grown on gold-film electrodes, into continuous readings of the electrodes’ electrochemical impedance. It was our objective to directly observe and compare cellular behaviors before and after electric field mediated delivery of different NA species by impedance measurements. In this concept, the gold-film electrodes serve both, (i) the application of elevated electric fields for delivery and (ii) non-invasive impedance monitoring of cell behavior. The latter is referred to as *electric cell-substrate impedance sensing* (or short ECIS) in the literature^11^.

Since its first introduction by *Giaever* and *Keese* in 1984^12^, ECIS has become a widely used, label-free analytical technique to monitor phenotypic assays with adherent cells. Due to the insulating properties of the plasma membranes, cells grown on gold-film electrodes act like dielectric bodies forcing alternating current (AC) below a threshold frequency to flow around them. Consequently, the measured impedance is determined by the geometry of the narrow, electrolyte-filled channels between (cell-cell contacts) and beneath the adherent cells (cell-matrix contacts). Thus, time-resolved impedance readings report on cell shape changes with a resolution below ordinary optical microscopes^10^. Based on its sensitivity, outstanding time resolution and throughput, ECIS allows monitoring a vast amount of different phenotypic assays. Combining microscopic inspection of cytoplasmic delivery with impedance-based monitoring of the cells before and after electroporation provides quantitative indicators for both, efficiency of delivery and invasiveness of the operation.

Previous studies have already combined *ISE* with impedance monitoring (i) to track the invasiveness of electroporation protocols and (ii) to detect phenotypic alterations following the introduction of bioactive molecules^10,13,14^. In this work we focus on the intracellular delivery of different species from the family of coding nucleic acids while the phenotypic consequences are monitored by impedance readings. This focus is motivated by the unparalleled capability of NAs to provide either persistent new *functions* to cells (gain-of-function, GoF) or persistently disable existing ones (loss-of-function, LoF) on various time scales. The terms *gain-of-function* or *loss-of-function* are used here in the broader biomedical sense that cell manipulation may *provide a new function* to the cell, which has not been there originally, or *disable an existing function*. By virtue of their molecular composition, the different NA species share a polyanionic nature but their molecular masses range from around 10^4^ g/mol for siRNA to more than 10^6^ g/mol for plasmid DNA. We demonstrate successful delivery of these various nucleic acid species using optimized electroporation parameters.

Assays as presented here will add a versatile analytical approach to the laboratory toolbox used in basic as well as applied research across biomedicine or biotechnology in particular since (i) *ISE* provides spatially confined delivery of NAs to subpopulations of a given cell monolayer simply defined by the geometry of the electrodes; (ii) ECIS and *ISE* are based on thin-film electrodes, so that they are easy to integrate in *organ-on-chip* (OoC) devices and (iii) *ISE* is minimally invasive and it may serve as a gentle means to manipulate even very sensitive and fragile cell types. Integration of *ISE*-ECIS may pave the way to more advanced *microphysiological systems* (MPS) in the near future^15,16^.

## Materials and methods

### Cell lines

Human Embryonic Kidney 293T (HEK293T; provided by the lab of A. Buschauer, Universitaet Regensburg, G) cells were cultivated in Dulbecco’s Modified Eagle Medium with 4.5 g/L D-glucose (DMEM, Sigma) supplemented and 10% (v/v) fetal bovine serum (FBS, Thermo Fisher), 2 mM L-glutamine (Sigma) and 100 µg/mL penicillin/streptomycin (Sigma). Wilde-type Chinese Hamster Ovary (CHO-K1) cells and CHO-EGFP cells, stably transfected to constitutively express EGFP, were cultivated in Ham’s F-12 medium supplemented with 10% (v/v) FBS, 100 µg/mL penicillin/ streptomycin and 2 mM L-glutamine. To establish continuous selection pressure, the culture medium for CHO-EGFP cells was supplemented with 400 µg/mL G418 (Invitrogen). Both cell lines were provided by the lab of A. Goepferich and M. Breunig, both affiliated with Universitaet Regensburg (G). Normal rat kidney (NRK, strain E52; purchased from DSMZ GmbH, Braunschweig, G) cells were cultivated in DMEM with 3.7 g/L NaHCO_3_ and 4.5 g/L D-glucose (Sigma) supplemented with 5% (v/v) FBS, 100 µg/mL penicillin/streptomycin and 2 mM L-glutamine. HaCaT cells (purchased from Cell Line Services GmbH, Eppelheim, G) were routinely grown in DMEM with 3.7 g/L NaHCO_3_ and 4.5 g/L D-glucose (Sigma) supplemented with 10% (v/v) FBS, 100 µg/mL penicillin/streptomycin and 2 mM L-glutamine. All cell lines were cultivated at 37 °C in a humidified incubator in a 5% (v/v) CO_2_ atmosphere. NRK and HaCaT cells were sub-cultivated once, HEK293T, CHO-K1, CHO-EGFP cells twice a week at cell confluences of around 100% (NRK cells) or 80% (HaCaT, HEK293T, CHO-K1 and CHO-EGFP cells).

### Preparation of pDNA and mRNA encoding green fluorescence protein (GFP)

Plasmid DNA (pDNA) encoding EGFP (enhanced green fluorescence protein) within the pFLAG-CMV-4 vector backbone was prepared using a Qiagen^®^ Maxi Prep kit following the manufacturer’s protocol. The vector backbone was a gift from Judy Lieberman (Addgene plasmid #80951^17^.

For in vitro transcription of *superfolder (sf) EGFP* mRNA, Biocat GmbH (Heidelberg, Germany) synthesized a DNA template containing the sf EGFP gene with an upstream T7 promoter sequence and a downstream Poly (A) tail (100mer). In vitro transcription was conducted using this DNA template at a concentration of 60 ng/µL. The DNA template was mixed with (5x) nucleotide mixtures including ATP, CTP and UTP (final concentration f.c. 18.75 mM), GTP (f.c. 7.5 mM), 5´ Cap analogue (f.c. 2.5 mM), (5x) transcription mixture including MgCl_2_ (f.c. 75 mM) and Spermidin (f.c. 0.5 mM), (20x) enzyme mix containing T7 polymerase (f.c. 1 U/µl). The completed reaction mixture was incubated over night at 37 °C. Resulting mRNA was purified by gel filtration using DyeEx2.0 spin columns (Qiagen) or NAP5 columns (Cytiva). mRNA concentrations were estimated using the Nanodrop (Nanodrop 2000 c) spectrophotometer by measuring the absorbance at 260 nm. Concentrated mRNA samples were prepared using the Eppendorf concentrator 5301.

### Fluorescence microscopy

Fluorescence imaging was conducted using an upright confocal laser scanning microscope (CLSM; Nikon Eclipse 90i, Japan). Cells on electrodes were imaged either with a water-immersion objective (NIR Apo 60×/1.0 W) immersed in pre-warmed buffer or a dry objective (Plan Fluor 10×/0.30 W). The confocal pinhole diameter was set to 30 μm (‘small’). Green fluorescence was excited at 488 nm and detected using a band pass filter BP500-530 nm, while red fluorescence was excited at 543 nm and detected using a long pass filter LP650 nm. Detector gains were typically set to 100–120 for 488 nm excitation and 120–130 for the 543 nm excitation. Images acquired with the 10× objective were digitally magnified threefold for improved visualization.

### *In situ electroporation* and impedance monitoring

In ECIS, adherent cells cultured to confluence on pairs of planar gold-film electrodes are continuously monitored by non-invasive and time-resolved measurements of the electrode’s electrochemical impedance. These planar electrodes, deposited upon the bottom of multi-well plates, are electrically connected via the overlaying electrolyte (culture medium, buffer). As cells adhere to and spread across the electrode surface, their presence results in progressively increasing electrode impedance. At lower frequencies (< 10 kHz), the current primarily crosses the cell layer on paracellular pathways around the cells, whereas at higher frequencies (< 10 kHz), capacitive coupling through the cell membranes becomes significant^11,18^. The AC frequency providing the highest sensitivity for cell morphology changes depends to some degree on the electrode area and the cell type under study. It is reported in the results section individually with every experiment. In all experiments we used non-invasive current and voltage amplitudes in the order of a few µA or mV, respectively. These conditions have proven not to harm the cells or affect their phenotype.

The term *electroporation* refers to the transient permeabilization of the cell membrane induced by short, high-intensity electric pulses^19^ forming transient, hydrophilic pores in the lipid bilayer. A cell-type specific transmembrane voltage of about 1 V must be induced by the external electric field to achieve a dielectric breakdown of the membrane resulting in pore formation^20–22^. This process allows for the intracellular delivery of extracellular molecules that are otherwise not membrane-permeable. They remain within the cytosol after membrane resealing within seconds after the electric pulse is terminated. Depending on the magnitude and duration of the applied pulse, electroporation can be *reversible* or *irreversible*. Electroporation has been applied for the delivery of both, small molecules and macromolecules across a wide range of cell types and species.

**Fig. 1:**
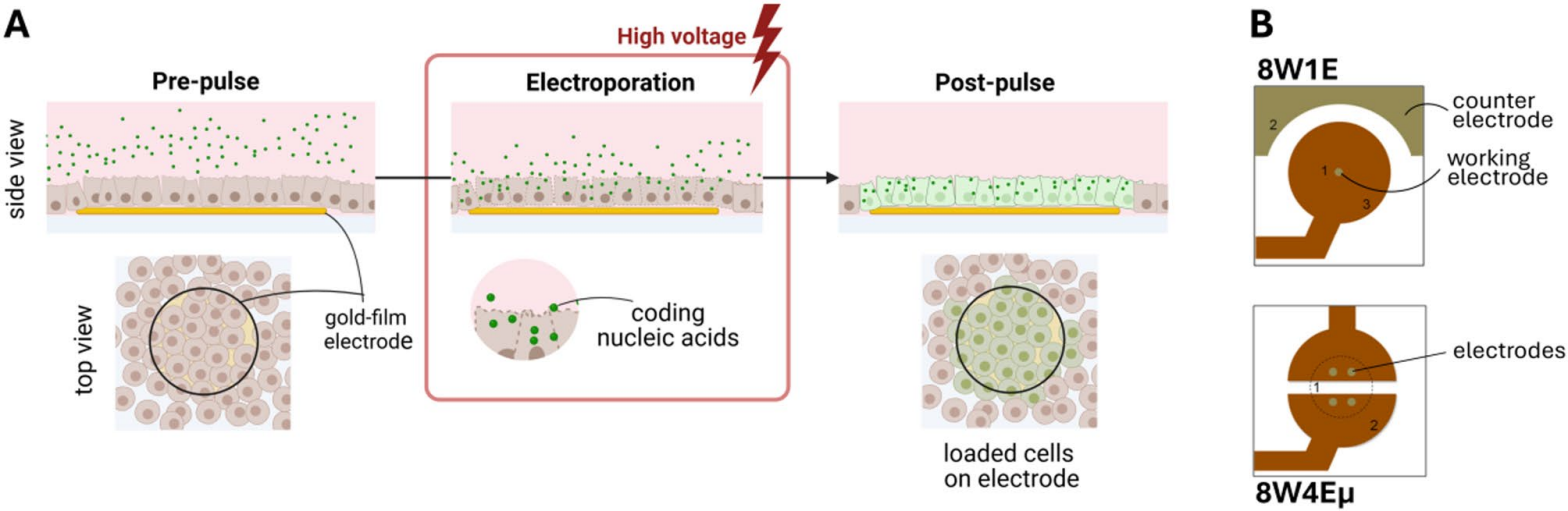
Concept of loading adherent mammalian cells with various types of coding nucleic acids (NA) by *in situ electroporation* (*ISE*) using 8W1E or 8W4Eµ electrodes. (**A**) Gold-film electrodes are covered with adherent cells. Cells are loaded with NAs of interest during one or more short AC voltage or current pulses. Before pulse application (pre-pulse), cell membranes are impermeable to NA. The latter are present only in the extracellular buffer. During *ISE*, cell membranes open transiently, NAs are allowed to enter the cytosol. Only cells in direct contact with the electrodes get electroporated. Cells residing on the insulating polymer around the electrodes are not affected by the electric pulse. After the electroporation pulse, membranes reseal and NAs are captured inside the cytosol. (**B**) Two types of electrodes were used in this study. 8W1E electrode arrays consist of 8 wells containing one small working electrode (1) defined by a circular opening in an insulating polymer layer (3) and a bigger counter electrode (2). Each well of 8W4Eµ electrode arrays includes four small gold electrodes (1) acting in pairs as working and counter electrodes. The active electrode area is defined by four circular openings in the insulating polymer layer (2).

In contrast to conventional electroporation applied to cells in suspension, *ISE* involves cells that are cultured to confluence on a conductive substrate used as electrode. The cells remain adherent during exposure to the electric pulses. Under these conditions, membrane proteins, the cytoskeletal filament network, cell-cell and cell-substrate contacts are fully functional, supporting membrane resealing and functional recovery after transient membrane permeabilization^23^. The integration of *ISE* into the ECIS methodology enables the concurrent application of electroporation pulses while allowing for real-time impedance monitoring (i) membrane recovery dynamics and (ii) the cellular responses to the delivery of extracellular solutes into the cytoplasm^9^. All experiments in this study were conducted using 8-well electrode arrays (well volume 400 µl) that come with different electrode configurations as specified below (cf. Fig. 1). Electrode arrays were purchased from Applied BioPhysics Inc. (Troy, NY). This study relies on two different electrode arrays indicated as 8W1E or 8W4Eµ. 8W1E arrays have eight wells (**8W**1E) with one circular working electrode of 250 μm diameter (8W**1E**) and a much bigger counter electrode (CE) per well. 8W4Eµ arrays consist of eight wells (**8W**4Eµ) with four circular electrodes of 250 μm diameter (8W**4E**µ) in each well but no counter electrode. Instead, sets of two circular electrodes are on the same gold film so that they are effectively in parallel. The second set with two circular electrodes is prepared on a separated gold-film and is arranged in series to the former (cf. Figure 1). In the 8W4Eµ layout, these two sets behave *formally* like working electrode and counter electrode even though they are of the same kind. They are spatially very close together but electrically not connected. Since WE and CE are close together in 8W4Eµ, we have previously described^14^ that it is possible to glue a silicon ring (diameter smaller than well size) around these four electrodes to reduce the working volume (8W4E**µ**) down to 30–50 µL which is not possible with 8W1E. Please note, 8W1E and 8W4Eµ behave electrically identical since each individual circular electrode is 250 μm in diameter. Placing two of these in parallel, in series to another set of two parallel equally sized electrodes produces electrically the same impedance even though the number of electrodes is different. Details of this micro-*ISE* have been described previously^14^.

Prior to cell seeding, electrode arrays were sterilized via argon plasma treatment and pre-conditioned with cell culture medium. For cell lines exhibiting weak substrate adhesion (HEK293T), the wells were additionally coated with crosslinked gelatin. Briefly, a 0.5% (w/v) gelatin solution (Sigma-Aldrich) prepared in water was added to the wells and incubated for 1.5 h at room temperature. Crosslinking was performed using 2.5% (w/v) glutardialdehyde (Sigma-Aldrich) in water for 10 min at RT, followed by thorough washing (10 ×) with water to remove residual cytotoxic aldehydes. Generally, cells were seeded directly onto the gold electrodes 24 h prior to electroporation for HEK293T and CHO cells, while HaCaT and NRK cells were seeded 48 h prior to electroporation to ensure confluence and adhesion. Before electroporation, the culture medium was gently aspirated and replaced with electroporation buffer EBSS^++^ (Eagle’s Balanced Salt Solution supplemented with 1 mM Ca^2+^ and 0.5 mM Mg^2+^) supplemented with the respective test molecules in concentrations that are detailed individually in the results section. All media and buffers share a physiological salt concentration showing a more or less constant conductivity of about 16 mS/cm.

### Repeated electroporation pulses

To improve delivery efficiency, repeated electroporation pulses were applied to a cell population under test in trains of three consecutive pulses using the same pulse parameters (AC frequency, amplitude, duration) with recovery phases of 10 to 30 min in between. To verify improved delivery efficiency for repeated electroporation pulses, NRK cells were grown to confluence on the electrodes and electroporated in presence of 100 µM propidium iodide (PI) in EBSS^++^. This red fluorescent dye is membrane-impermeable. It only emits fluorescence once it has intercalated into double stranded DNA. PI has been used in numerous electroporation studies as a electroporation reporter dye^24,25^. Impedance was recorded at 4 kHz before, after and in between the pulses (40 kHz, 4 V_rms_, 200 ms) to monitor the recovery of the cells. Microscopic inspection of the electroporated cells and their PI loading was conducted by CLSM directly after pulsing and impedance monitoring were terminated. Parameters of microscopic imaging are detailed above.

### Electroporation in presence of plasmid DNA or mRNA

HEK293T cells were seeded at a density of 250.000 cells/cm^2^ in a volume of 400 µL per well in 8well electrode arrays (Applied BioPhysics Inc. (USA), type 8W1E; see Fig. 1 for electrode layout; well area is always 0.8 cm²). Cells were allowed to grow for 24 h before electroporation to ensure adherence and confluence. Following medium removal, cells were bathed in 100 µL of electroporation solution containing either EGFP-encoding plasmid DNA (7000 base pairs, final concentrations: 25, 50 and 100 µg/mL) or GFP-encoding mRNA (3000 bases, final concentrations: 300, 500 and 1000 µg/mL) both prepared in EBSS^++^. The electrode arrays were then connected to an ECIS-ZΘ or ECIS-1600R measurement platform (Applied BioPhysics Inc.) inside regular cell culture incubators. Time-resolved impedance measurements were started soon after using an AC frequency of 40 kHz. Cells were allowed to equilibrate for 30 min prior to the application of the electroporation pulse. While the ECIS-1600R device uses voltage pulses for membrane electroporation, the ECIS-ZΘ uses current pulses. We found both approaches to provide very similar outcomes when the corresponding amplitudes are carefully adjusted. Pulse duration and frequency of the AC pulses were constant for both devices. Electroporation was carried out by applying an alternating voltage pulse of 5 V amplitude using ECIS-1600R or an alternating current pulse of 2.2 mA using ECIS-ZΘ, both at an AC frequency of 40 kHz and for a duration of 200 milliseconds. Control cells were incubated with either 50 µg/mL plasmid DNA or 1000 µg/mL mRNA but were not exposed to the electric pulse. Approximately 15 min after electroporation, 300 µL of fresh culture medium was added to each well. Cells were subsequently monitored for 24 h post electroporation, after which EGFP expression was assessed via confocal fluorescence microscopy (see above).

### Delivery of different siRNA species by electroporation

1. *Transfection indicator (siGLO Red*^*™*^***)***: Experimental protocols for delivery of siRNA were optimized using the fluorescence transfection indicator *siGLO Red*^*™*^ to allow for microscopic evaluation of loading efficiency and intracellular localization. The double-stranded RNA carries a Thermo Scientific^™^ NuLight^™^ DY-547 fluorophore (Dharmacon^™^). Stock solutions of 20 µM *siGLO Red*^*™*^ in 1× siRNA buffer (Qiagen) were stored at − 20 °C.
2. *Small interfering RNA targeting EGFP (siRNA*^*EGFP*^***)***: Sequence-specific knockdown of EGFP expression was performed by the electroporation of small interfering RNA (siRNA) targeting the EGFP mRNA using sense (GAA CUU CAG GGU CAG CUU GCC G) and antisense (GCA AGC UGA CCC UGA AGU UCA U) strands, containing 22 nucleobases each. Stock solutions of 100 pmol/µL were stored at − 20 °C. Both complementary strands were annealed before use to get a stable double-stranded siRNA. For annealing, 15 µL of sense RNA was mixed with 15 µL antisense RNA and 10 µL 10× annealing buffer (100 mM TRIS-hydrochloride, 1 M sodium chloride and 10 mM ethylene-diaminetetraacetic acid, pH = 7.5 with 1 M sodium hydroxide) and 60 µL RNase-free water. The annealing process was started by heating the solution to 70 °C for 10 min in a Thermocycler.
3. *Small interfering RNA inducing cell death (siRNA*^*cell-death*^*)*: The knockdown of genes required for cell survival was demonstrated by using commercial *cell death siRNA*. *AllStars Mn/Rn Cell Death Control siRNA* (Qiagen) was provided as annealed double-stranded oligonucleotide and was dissolved to a concentration of 10 µM in sterile RNase-free water and stored at − 20 °C.
4. *Small interfering RNA with random sequence (siRNA*^*scr*^*)*: Double stranded non-targeting scrambled siRNA served in all experiments as control. AllStars Negative Control siRNA (Qiagen) was purchased in an annealed, double-stranded, and unmodified form. It was stored with a concentration of 20 µM in RNase-free water at − 20 °C.

For electroporation of siRNA, CHO-EGFP cells were seeded on 8W4Eµ electrode arrays (cf. Fig. 1) in a density of 80.000 cells/cm^2^ 24 h prior to adding the electroporation solutions. Different concentrations of the individual siRNA species were prepared in EBSS^++^. Before impedance measurements were started, these solutions were added to the cells in a nuclease-free environment. After baseline recording, *ISE* (40 kHz, 4 V_rms_, 500 ms) was applied three times successively. 400 µL of complete medium was applied to the wells after recovery from the final electric pulse as revealed by impedance readings. Delivery of siRNA was examined microscopically 24–72 h after pulse application as described above.

### Electroporation of aptamers

To study aptamer delivery in general, we selected a DNA aptamer as a probe with a random sequence, addressing no intracellular target but carrying a green fluorescent label for localization inside the cells. The aptamer with the sequence 5’- AAG ATA CGC GTC TCC AGG GAT AAA TGA GGA CGA CCG CAG CGC GTG CGA GG − 3’ was coupled to a fluorescent modification (FAM-EX-5) at its 5’ end. Aptamers were dissolved in TE-buffer (10 mM Tris, 1 mM EDTA, pH 8) and stock solutions of 100 µM were stored at − 20 °C. 8W4Eµ arrays (cf. Fig. 1) were modified with small silicone rings (Ø: 5 mm) glued into the wells to confine the working volume^14^. Three mammalian cell lines were included in this study: CHO K1, HaCaT, and NRK cells. Electroporation solutions were prepared by diluting a 100 µM stock solution of aptamers in EBSS^++^, yielding a final concentration of 15 µM in a total volume of 40 µL per well. Following the application of the aptamer solution to confluent cell monolayers, the cells were allowed to equilibrate within the measurement chamber for approximately 10 min. Repeated electroporation pulses were then applied in trains of three individual pulses (CHO K1: 40 kHz, 4 V_rms_, 500 ms; HaCaT: 40 kHz, 5 V_rms_, 500 ms; NRK: 40 kHz, 4 V_rms_, 200 ms). After a recovery period of 45 min, cells were gently washed with PBS^++^ (Phosphate Buffered Saline supplemented with calcium and magnesium) prior to microscopic analysis.

As a control for electroporation-mediated delivery, additional cell samples were chemically fixed and permeabilized for subsequent loading of the cytosol by a 15 µM aptamer solution. Fixation of cellular monolayers was achieved by incubation with paraformaldehyde (4% (w/v) in PBS^++^) for 10 min at room temperature. After the paraformaldehyde solution had been aspirated, a solution of 0.2% (v/v) Triton-X-100 detergent in PBS^++^ was added for membrane permeabilization. Followed by a 10-minute incubation period, the detergent was removed, and cells were incubated with the aptamer solution. Before microscopic inspection (see above), cells were washed once.

### Chemical transfection

Chemical transfection served as an independent control to electroporation with respect to delivery efficiency and gene expression. Generally, transfection was performed according to the protocols provided by the manufacturers of the different transfection reagents. In brief, cells were grown to subconfluence (70−90% confluency) prior to chemical transfection. Two solutions were prepared separately: (i) transfection reagent and (ii) nucleic acid diluted in buffer. Both solutions were combined immediately before they were added to the cells. Final concentrations and volumes are listed in Table 1. Transfected cells were examined using confocal laser scanning microscopy after an incubation period of 24 h under normal cell culture conditions. Details of microscopic analysis are mentioned in the corresponding paragraph above.

**Table 1:** List of transfection reagents with their final (undiluted) volume per well, the nucleic acid preparations with their final concentrations in the well and the buffers that were used in the different chemical transfections of plasmid DNA, mRNA, and siRNA as used in this study.

Nucleic Acid (NA)	Transfection Reagent (TR)	Buffer	Final Volume of undiluted TR added per Well	Final NA concentration in well	Total Volume in well (cell culture medium)
Plasmid DNA	Transporter 5^®^ polyethylenimine MAX (polysciences)	DMEM-high glucose	1.5 µL 8-Well Array (0.8 cm^2^)	2.5 µg/mL	400 µL
mRNA	Lipofectamine^®^ MessengerMAX	OPTI-MEM	1.5 µL 8-Well Array (0.8 cm^2^)	2.5 µg/mL	400 µL
siRNA	Lipofectamine^®^ RNAiMAX	OPTI-MEM	1.2 µL 8-Well Array (0.8 cm^2^)	25–200 nM	400 µL

### Evaluation of impedance and microscopic data

Impedance time courses were normalized by dividing the individual impedance readings by the value of the last data point before cell manipulation (set to 1). Normalized impedance was then plotted as a function of time. Microscopic images were analyzed by comparing the fluorescence intensity of cells residing on the electrodes and electroporated with different types of nucleic acids to cells on electrodes pulsed only in presence of buffer. This image analysis was conducted using *ImageJ*. The average pixel brightness was measured and calculated for selected regions of interest (ROI). Integrated fluorescence intensity within the ROI was divided by the number of contributing pixels.

## Results and discussion

### Delivery of propidium iodide into adherent NRK cells by repeated electroporation pulses

Besides their sequence, intracellular manipulation by coding nucleic acids (NA) relies to a large extent on the amount of NA injected into living cells. We have shown previously that it is possible to deliver plasmid DNA into adherent cells by *ISE*^11^. In this study, we followed the concept of applying repeated electroporation pulses to the same cell population to provide overall more time for the extracellular NAs to get across the permeabilized membrane and to increase the amount of exogenous NA inside the cell. This concept requires the impedance of the membrane to be restored between the individual pulses as the voltage is otherwise not efficiently delivered to the membrane but to the electrodes or the buffer^10^. To establish and verify the benefit of repeated electroporation pulses, we used a simple, well-established and membrane-impermeable fluorophore as an indicator for intracellular loading: red-emitting propidium iodide (PI) with a molecular mass of 680 g/mol. Once PI gets access to the cytosol, it diffuses readily into the nucleus via the nuclear pore complex, intercalates into the DNA and is thereby trapped inside the cell even if the electroporation pulse is irreversible. PI does not show any significant fluorescence without intercalation in double stranded DNA. In these experiments, confluent NRK cells were pulsed repeatedly once, twice or three times using pulse parameters that have been optimized for single pulse electroporation before (40 kHz, 4 V_rms_, 200 ms). The experiment also included a control with cells not being electroporated at all but incubated with PI solution in the same concentration that was used in the electroporation experiments. Impedance was recorded before and after the individual pulses (EP 1, EP 2 and EP 3).

**Fig. 2:**
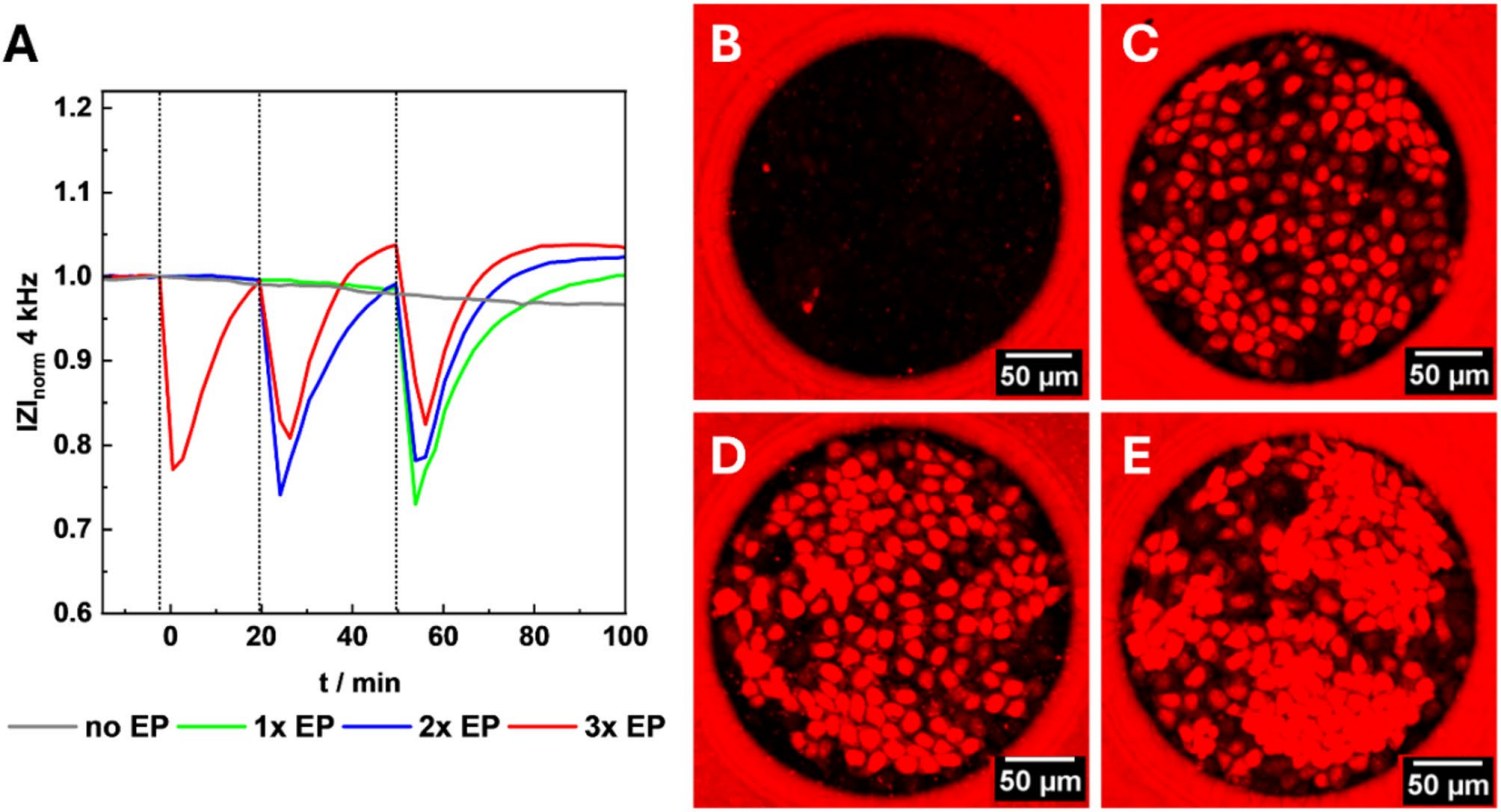
Confluent NRK cells exposed to repeated electroporation pulses in presence of 100 µM propidium iodide. (**A**) Typical time course of the normalized impedance of 8W4Eµ electrodes (cf. Fig. 1) covered with NRK cells during the application of one, two or three sequential electroporation pulses indicated by the dashed lines. Grey: no electroporation; green: 1 × electroporation; blue: 2 × electroporation; red: 3 × electroporation with 4 V_rms_ at 40 kHz for 200 ms. Fluorescence micrographs were taken 30 min after the third electroporation pulse using confocal laser scanning microscopy (**B**: no electroporation, **C**: 1 × electroporation, **D**: 2 × electroporation, **E**: 3×electroporation).

Figure 2A shows typical time courses of the normalized impedance during repeated *ISE* of NRK cells. After every individual pulse, the normalized impedance (4 kHz) decreased immediately and returned to baseline (pre-pulse) levels within 20–30 min indicating full recovery of the cell layer from the electroporation event. CLSM micrographs (2**B**–**E**) demonstrate the loading efficiencies provided by the different numbers of pulses applied. Without electroporation pulse (**B**), PI is unable to diffuse across the cellular plasma membrane. For cells pulsed once (**C**), a clear uptake of the fluorescence probe was observed from the intense nuclear staining originating from PI intercalation into chromosomal DNA. It is notable that repeated pulse application with the same pulse parameters once or twice led to a more intense red fluorescence within the cells on the electrode surface (**D**, **E**) verifying an improved loading efficiency. The insulating polymer that was used to delineate the circular electrodes by photolithography shows a strong red autofluorescence. This polymer is covering the surface across the entire well except for the active electrodes where it has been removed during production. Presence of the polymer in the periphery of the electrodes is responsible for the strong red fluorescence emission which does, however, not affect analysis of cells on the electrodes. Microscopic documentation of cells on the electrodes revealed a progressive increase in intracellular fluorescence with each additional pulse, indicating a cumulative uptake of PI. Real-time monitoring of cell morphology before and after the individual pulses ensured sufficient recovery time between the pulses to maintain cell viability. Previous studies have demonstrated that repeated electroporation in presence of fluorescent, membrane-impermeable tracers improved the delivery efficiency to suspended cells^26–28^. This concept from suspension electroporation proved to be a very useful strategy to improve *ISE* delivery of extracellular material into the cells - or release of intracellular material out of the cells dependent on the assay. Electroporation requires rather high extracellular concentrations of the solute to be delivered as the membrane is only open during the electrical pulses and shortly beyond. Typically, plasma membranes reseal within less than a second after pulse application in our setup^9^. The time span of membrane permeabilization defines the time span available for solute diffusion from the extracellular space to the cytosol. Since interesting biomolecules capable of specifically manipulating adherent cells are precious and expensive, repeated *ISE* may offer a strategy to improve the delivery efficiency without increasing extracellular concentrations. Once several working electrodes are placed side-by-side within one well, repeated electroporation with individual repeats allows delivering increasing amounts of solutes to the different cell populations from one working solution. So it may even serve as experimental workflow to conduct dose-response analyses of membrane-impermeable modulators of cell physiology, e.g. dose-dependent compound screening for electrochemotherapy in a single well. Next to repeated electroporation, the development of 8W4Eµ electrode arrays was another strategy to make *ISE* more cost-effectively. *ISE* using 8W4Eµ electrode arrays brings down the working volumes from 150 to 30 µL so that even delivery of antibodies or other recombinant proteins becomes realistic since *protein transfection* is a similarly challenging problem in biomedicine. Even though it does not aim for cytosolic loading, repeated pulse application is also useful with respect to fine tuning *irreversible electroporation (IRE).* IRE leads to spatially confined cell death within a cell monolayer and it has become a clinical strategy for cancer treatment^29^. As such it may benefit from *in vitro* studies.

### Electroporation-mediated delivery of aptamers into various cell types

**Fig. 3:**
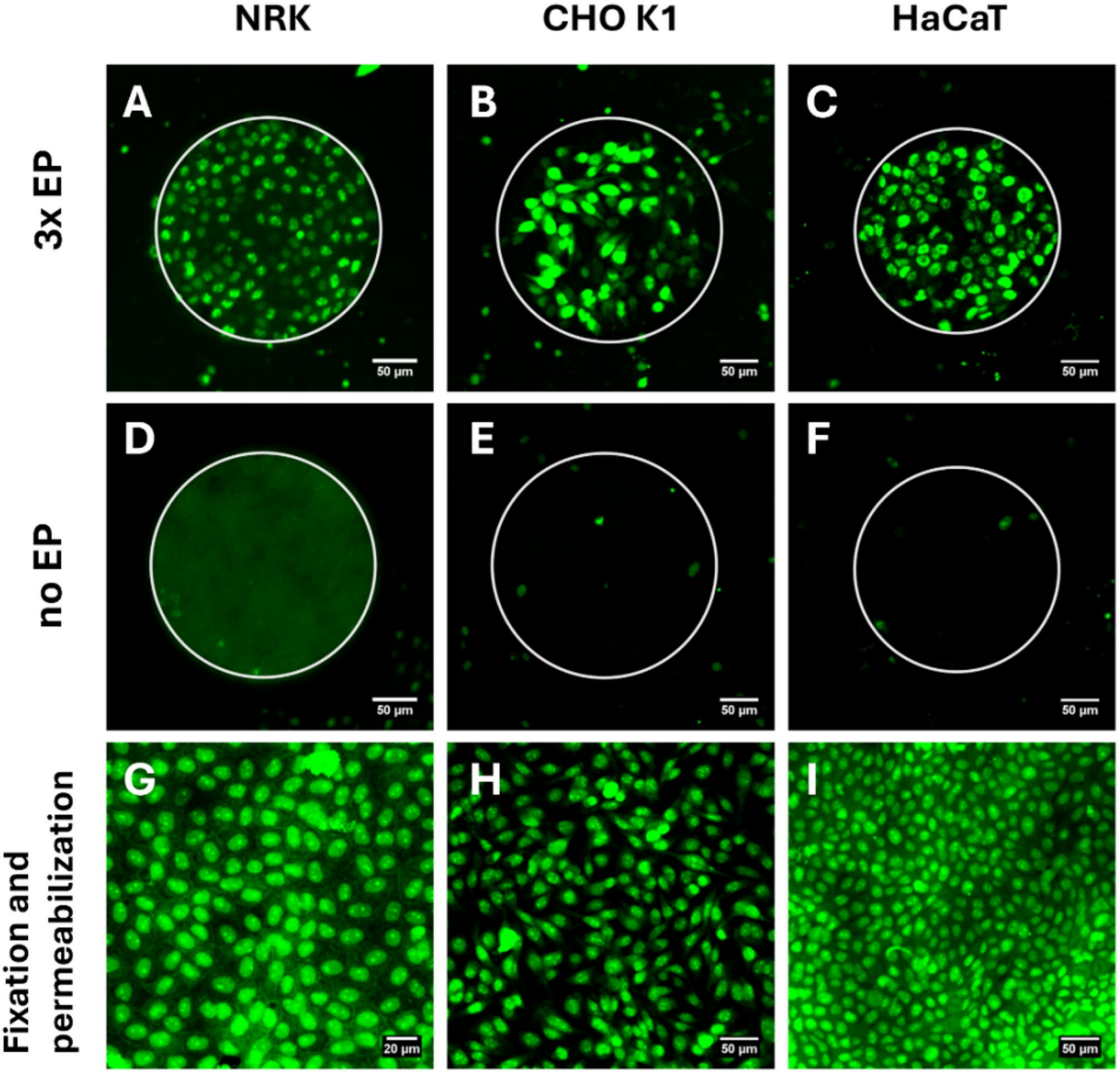
Overview of aptamer delivery into three cell lines by *ISE*. Confocal fluorescence micrographs of NRK (**A**), CHO-K1 (**B**) and HaCaT cells (**C**) in presence of 15 µM fluorescently labeled tracer aptamer after three electroporation pulses (CHO K1: 40 kHz, 4 V_rms_, 500 ms; HaCaT: 40 kHz, 5 V_rms_, 500 ms; NRK: 40 kHz, 4 V_rms_, 200 ms; 8W4Eµ electrode array) or without electroporation (**D**-**F)**. Loading of the same cell lines after fixation and chemical membrane permeabilization (**G**-**I**).

Aptamers are short, single-stranded nucleic acids (DNA or RNA) with the capacity to selectively bind their target within rather complex matrices. They are capable of molecular recognition similar to antibodies but they are entirely synthetic, chemically more stable and of significantly lower mass than antibodies. Accordingly, aptamers are a very interesting class of molecules for specific intracellular manipulation of cells by binding and potentially blocking their target’s function inducing phenotypic changes. But similar to other NAs, the multiple negative charges of aptamer molecules create a challenge to effectively transfer the molecules into cells. *ISE*-based delivery of aptamers was established in this study for NRK, HaCaT and CHO K1 cells using a fluorescently labeled DNA-aptamer consisting of 50 deoxynucleotides (molecular mass is app. 17 kDa) with randomized sequence as tracer compound. It does not target any intracellular structure but is rather used as a molecular probe. Cells were grown to confluence on gold-film electrodes (Applied BioPhysics Inc., type 8W4Eµ, cf. Figure 1) prior to their exposure to the aptamer solution (15 µM in EBSS^++^). Intracellular aptamer delivery was conducted by three consecutive electroporation pulses as described in the preceding section. Parameters of the individual pulses within the set of three were kept constant but were individually adjusted for the different cell types with respect to frequency, amplitude and duration according to preceding dye loading experiments. Impedance was recorded before and after the individual pulse applications. Thus, cell recovery was monitored after every pulse. Aptamer loading efficiency was evaluated approximately 1–1.5 h after pulse application and compared to cells that were chemically permeabilized and exposed to the same aptamer solution. Figure 3 summarizes the outcome of the two delivery strategies (*ISE* versus chemical permeabilization) for three cell lines.

Fluorescence micrographs prove the presence of the fluorescently labeled aptamers inside the cells after electroporation and membrane resealing. Loading efficiency after multiple electroporation pulses (upper row, **A**–**C**) is compared to cells exposed to the same aptamer solution without any electroporation (middle row, **D**–**F**). The circular electrode border is marked as a white circle. Cells located within the electrode area in A-C show green fluorescence. In contrast, cells residing next to the electrode not experiencing an electroporation pulse do not show green fluorescence after washing off all extracellular aptamers. Thus, membrane electroporation is needed for successful delivery. The micrographs confirm that all cell types have been successfully loaded with the fluorescent aptamers. Closer inspection of the micrographs reveals nuclear accumulation of the aptamers even though they were not designed to target nuclear structures. Co-localization of aptamers and lysosomes using LysoTracker^®^ (cf. supporting information SI 1) showed no detectable overlap, indicating predominant localization of aptamers in the nucleus. We suppose that the aptamers freely diffuse into the nucleus via the nuclear pore complex (NPC) and bind to the positively charged histone proteins. When cells were fixed and chemically permeabilized prior to aptamer exposure, we observed a similar distribution of aptamers inside the cells (lower row, **G**–**I**) with significant accumulation in the nucleus. A similar observation has been made by others before, who studied the intracellular uptake of aptamers into cells with compromised plasma membranes in MCF-7 cells. The intracellular binding is presumably unspecific but not yet studied in detail^30^.

Taken together, it is feasible to specifically manipulate adherent cells by time- and spatially controlled intracellular delivery of aptamers, for instance, to interfere with (i) protein-protein, (ii) protein-nucleic acid or (iii) ligand-receptor interactions and to monitor the associated *gain-of-function* or *loss-of-function* by impedance analysis. As the delivery is spatially confined, it will be possible to modulate precisely defined subpopulations of the cell layer on the electrode while cells in the periphery of the electrode are not manipulated. So it is possible to study manipulated cells and controls side-by-side within the same well or within the same microfluidic channel which is impossible using chemical transfection. This option may provide enormous advantages for work with organ-on-chips in which different cells are spatially separated but exposed to the same extracellular buffer. In combination with repeated, dose-dependent loading several new experimental strategies open up for OoC- and MPS-systems.

### Localized Delivery of Plasmid-DNA to HEK293T Cells by *ISE*

**Fig. 4:**
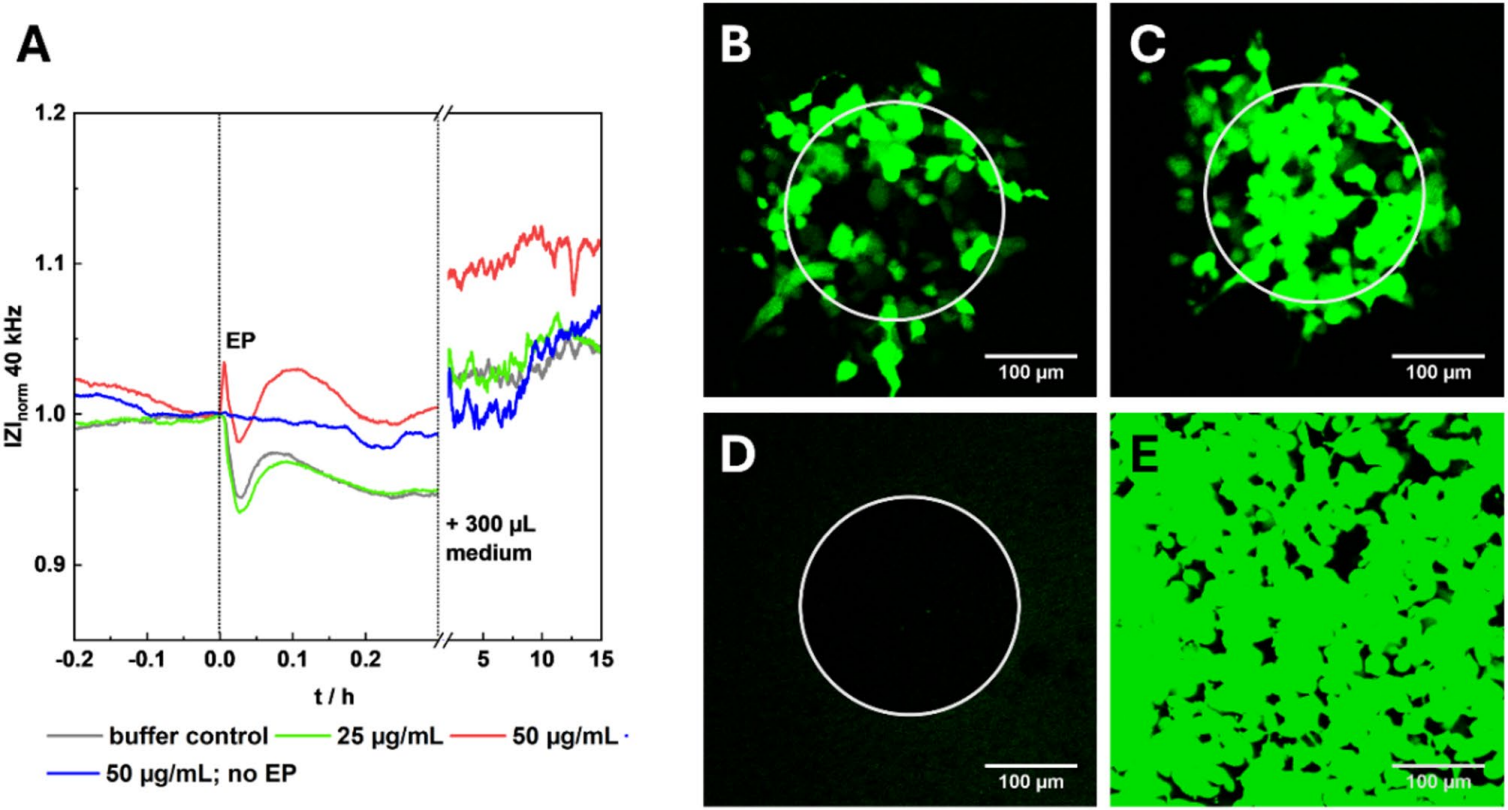
ISE of HEK293T cells in presence or absence of plasmid DNA encoding EGFP. (**A**) Typical impedance time courses of HEK293T cells when exposed to an electroporation pulse (40 kHz, 200 ms, 5 V_rms_) at t = 0 h in presence (green, red) or absence (grey) of pDNA. Cells were seeded 24 h prior to electroporation on 8W1E electrode arrays. **Grey**: buffer (EBSS^++^), electroporation; **green**: 25 µg/mL pDNA, electroporation; **red**: 50 µg/mL pDNA, electroporation; **blue**: 50 µg/mL pDNA, no electroporation. Impedance was recorded at 40 kHz; each curve represents one well. (**B**–**D**) Fluorescence micrographs 24 h after the electroporation pulse. (**B**): 25 µg/mL pDNA, electroporation; (**C**): 50 µg/mL pDNA, electroporation; (**D**): 50 µg/mL pDNA, no electroporation. White circles delineate the electrodes used for pulse application. (**E**) Chemical transfection of HEK293T cells with the same pDNA encoding EGFP. Microscopy was conducted 24 h after transfection. CLSM images were recorded using an excitation wavelength of 488 nm.

This section addresses the heterologous expression of proteins in the cells of interest by intracellular delivery of the corresponding gene as plasmid DNA (pDNA). The idea of this assay is to induce expression of the gene of interest and monitor the impact of the corresponding protein on the cellular phenotype by impedance recordings. To investigate the loading efficiency of pDNA when *ISE* is used for membrane permeabilization, HEK293T cells were seeded on gold-film electrodes (8W1E, cf. Fig. 1) and received electroporation pulses in presence of varying concentrations of an EGFP-encoding plasmid (7 kbp; ~4.620 kDa) as an easy to detect model system. Typical time courses of the normalized impedance are shown in Fig. 4A.

HEK293T cells were monitored before and after a single electroporation pulse (40 kHz, 5 V_rms_, 200 ms, t = 0 h) to minimize invasiveness. Following a 30-minute post-pulse recovery period, electroporation buffer was replaced by serum-containing culture medium to allow for EGFP expression. Impedance was recorded over 15 h to assess the long-term, non-invasive nature of the electroporation process and EGFP expression. An immediate drop in impedance was observed for all cell populations receiving an electroporation pulse (red, grey and green curve), followed by impedance recovery to new baseline levels within 10–20 min. Cells that did not receive any pulse are unaffected (blue curve). Long-term impedance profiles for cells loaded with pDNA (green and red curves) were indistinguishable from controls that were either electroporated in absence of DNA (grey) or received no electroporation pulse in presence of pDNA (blue). Measurements confirmed the quick recovery and non-invasive nature of the electroporation procedure, as cells electroporated in presence of pDNA exhibited impedance profiles similar to those of untreated control cells. Cells electroporated with the highest amount of pDNA (50 µg/mL, red) induced slightly elevated impedance values compared to all other conditions. With respect to the pool of all our experiments, this slight offset does not represent a significant phenotypic change. Please note, the impedance time courses seem to become noisier during the long term observation after pulse application. However, this is mainly due to the different time scales of the pre- and post-pulse impedance data and not an indicator for different cell dynamics. EGFP expression was verified 24 h post-electroporation using confocal laser scanning microscopy (Fig. 4B–D). As expected, EGFP fluorescence was dependent on plasmid concentration (25 µg/mL in Fig. 4B µg/mL in Fig. 4C). Fluorescence microscopy revealed successful EGFP expression in cells located within and adjacent to the electrode areas. Specifically, 30% and 55% of the cells residing directly on the electrode surface exhibited EGFP fluorescence following electroporation with 25 µg/mL and 50 µg/mL, respectively. These results are consistent with previous findings demonstrating efficient plasmid transfection via electroporation under optimized conditions^14^. EGFP-expressing cells are residing within the electrode area and slightly beyond the electrode borders. We interpret the presence of fluorescent cells beyond the electrode area as an indicator for cell proliferation and migration within the 24 h post-pulse incubation. It is important to note, that transfection via *ISE* requires the cell layer on the electrode not to be finally dense. Mitosis is required to allow pDNA to migrate into the permeabilized nucleus of dividing cells for transcription and/or integration into the genome. On the other hand, pulse application is most efficient for confluent cells without significant parts of the electrode uncovered allowing for sneak currents. Accordingly, *ISE*-based transfection, as conducted here, is a trade-off between no open spaces on the electrode and but still room for cell doubling. It requires careful adaptation of a cell-type specific cell density on the electrode surface controlled by seeding density and pre-culture time. No fluorescence was detected in those cells that were incubated with pDNA but not exposed to an electroporation pulse (**D**). For comparison, chemical transfection using polyethyleneimine (PEI) and the same plasmid DNA resulted in EGFP expression across the entire field of view (**E**), while spatially confined expression was exclusive to cell layers locally permeabilized by electroporation. Thus, *ISE* provides an experimental means for spatially confined transfection of *gain-of-function*. The pattern of heterologous protein expression is simply defined by the electrode geometry and easily adaptable. The experimental options arising from localized delivery have been discussed above for aptamers and they apply to pDNA in the very same way. Compared to chemical transfection, the efficiency of *ISE* is rather moderate. We suppose that this lower efficiency is at least partly due to the intracellular processing of the pDNA prior to expression. It has been shown recently, that electroporation-mediated delivery of pDNA is not enabled by diffusion through pores in the membrane but rather by clustering of DNA on the membrane and its enabling destabilization by the electric field. The latter triggers membrane mediated internalization^31^. While chemical transfection reagents are designed to assist endosomal escape and nuclear import, the electroporation-mediated delivery operates without these helpful additives. Thus, lower protein expression levels are very likely due to this lack of chemical assistance during endosomal escape and nuclear import. In future studies we will address whether it is meaningful and purposeful to assist electroporation-based pDNA delivery by small amounts of transfection reagents. Nonetheless, the setup provides a unique tool to study localized *gain-of-function* experiments induced by heterologous protein expression in animal cells.

### Spatially controlled delivery of mRNA into adherent cells by *ISE*

**Fig. 5:**
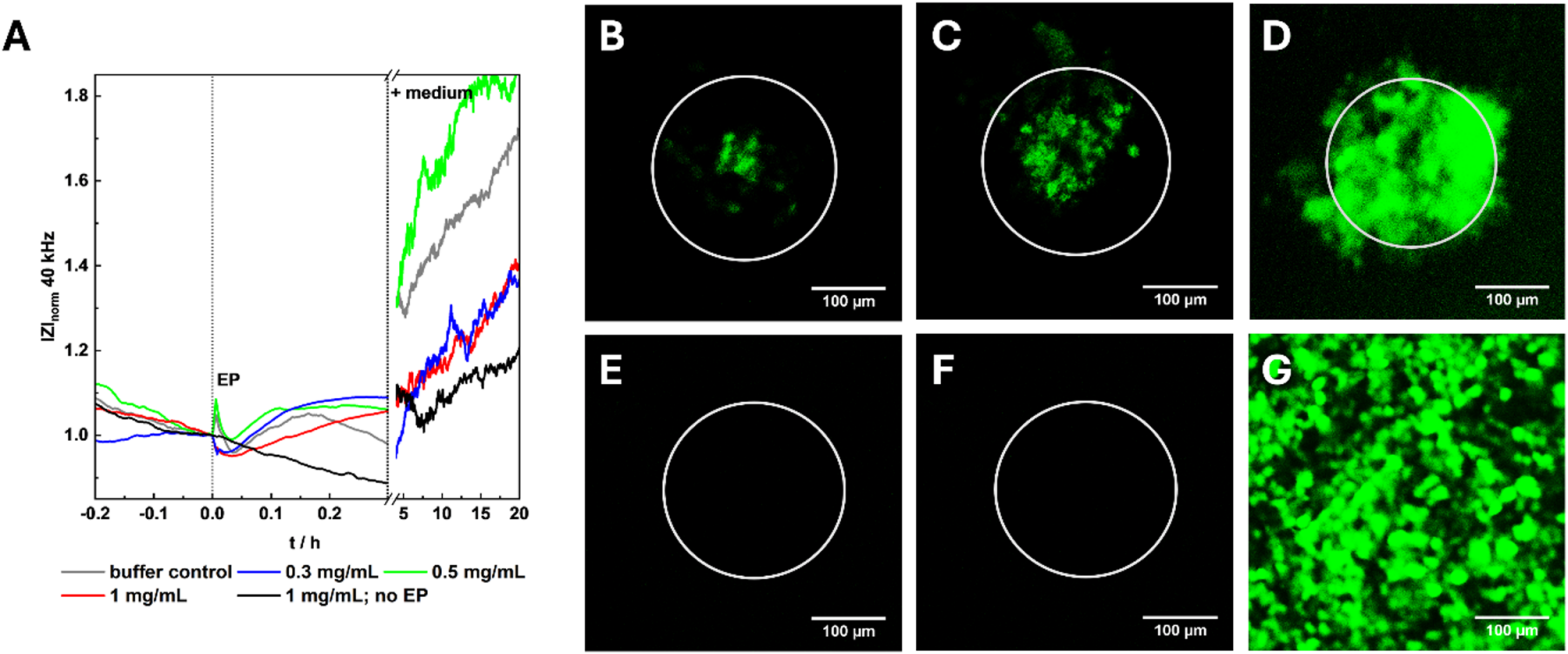
*ISE* of HEK293T cells in presence of EGFP-coding mRNA. (**A**) Impedance time courses of four individual cell populations. All populations were exposed to a single electroporation pulse (200 ms, 40 kHz, 2200 µA) at  t=0 h. Cells were seeded 24 h prior to electroporation on 8W1E electrode arrays. **Grey**: buffer (EBSS^++^), electroporation; **blue**: 0.3 mg/mL mRNA, electroporation; **green**: 0.5 mg/mL mRNA, electroporation; **red**: 1 mg/mL mRNA, electroporation; **black**: 1 mg/mL mRNA, no electroporation. Impedance was recorded at 40 kHz; each curve represents one well. (**B–G**) Fluorescence micrographs of differently treated cell populations monitored 24 h after the electroporation pulse. (**B**) 0.3 mg/mL mRNA, electroporation; (**C**) 0.5 mg/mL mRNA, electroporation; (**D**) 1 mg/mL mRNA, electroporation; (**E**) 1 mg/mL mRNA, no electroporation; (**F**) buffer control, electroporation. White circles indicate the borders of the small electrodes. (**G**) Chemical transfection with mRNA encoding EGFP. Microscopy of cells was conducted 24 h after transfection. Images were taken using the CLSM as described above using an excitation wavelength of 488 nm.

An alternative strategy to induce phenotypic changes in adherent cells by heterologous gene expression and to study them in situ by impedance measurements is the delivery of mRNA by *ISE*. To evaluate the delivery and expression efficiency of mRNA (3 kb; ~1.020 kDa), mRNA encoding EGFP was used as a model. The corresponding mRNA was synthesized from plasmid DNA in an in vitro transcription system and required only minimal purification prior to use, making it a rapid and efficient tool for transfection. To study mRNA delivery, HEK293T cells were cultured on circular gold-film electrodes (Applied BioPhysics, type 8W1E, cf. Fig. 1) and received electroporation pulses in presence of three different concentrations of this mRNA dissolved in EBSS^++^. Chemical transfection using *Lipofectamine MessengerMAX* served as positive control for *state-of-the-art* transfection efficiency. Impedance time courses throughout the experiment are presented in Fig. 5A. Impedance was continuously monitored before and after a single electroporation pulse (2200 µA, 40 kHz, 200 ms) to minimize invasiveness with long-term tracing extending to 20 h post-pulse.

As before, time courses of the normalized impedance show a transient decrease after application of the pulse at t = 0 h, followed by impedance recovery. This characteristic time course is indicative for membrane permeabilization, resealing and re-establishing the osmotic balance. 30 min post pulse, all curves returned to new steady-state values with a slight but unspecific downward trend across all conditions. Long-term monitoring revealed differential impedance dynamics: cells electroporated with 0.5 mg/mL mRNA (green) and the buffer control (grey) demonstrated the highest but obviously unspecific impedance increase compared to exposure to 0.3 mg/mL (blue) and 1 mg/mL (red). Fluctuations of the impedance signal, however, clearly indicate viability and metabolic activity of all cells independently from mRNA concentrations in the different electroporation solutions^32^. We therefore consider the differences in impedance time courses as insignificant and most likely due to slight differences in cell number. EGFP fluorescence was assessed 24 h after *ISE* by CLSM (**B**–**E**). Green fluorescence within the electrode area (white circle) confirmed successful delivery of mRNA and translation in a concentration dependent manner (**B**: 0.3 mg/mL, **C**: 0.5 mg/mL, **D**: 1 mg/mL). No fluorescence was detected in cell populations exposed to mRNA but not treated with an electroporation pulse (**E**) or when cells were loaded with electroporation buffer alone (**F**). In particular for mRNA concentrations < 1 mg/mL the fraction of EGFP positive cells was smaller than expected. Quantitative fluorescence analysis revealed EGFP expression in < 5% of cells at an mRNA concentration of 0.3 mg/mL. The fraction of expressing cells increased with increasing mRNA concentration to 15% for 0.5 mg/mL mRNA to 30% for 1 mg/mL. Higher expression efficiencies were anticipated based on prior reports, such as *Juncker et al.*^33^, and particularly the work of *Duckert et al.*, who reported up to 98% expression of mCherry in primary fibroblasts following mRNA electroporation^34^. In comparison, chemical transfection (**G**) revealed EGFP synthesis in more than 50% of the cells over the whole field of view without any spatial confinement. These outcomes were also lower than expected, particularly in light of previous findings by *Avci-Adali et al.*., who reported transfection efficiencies up to 95% in HEK293 cells using GFP mRNA in combination with Lipofectamine^®^2000^35^. At this point we can only speculate about the underlying reasons. However, since both delivery methods – *ISE* and chemical transfection–yielded lower transfection yields than expected, we take the outcome of the experiments as a robust indicator that *ISE* is well suited to deliver functional mRNA into adherent mammalian cells. As concluded for pDNA, *ISE* provides an experimental means for locally confined, heterologous protein expression in user-defined patterns and the ability to study the associated phenotypic changes by impedance analysis. As mRNA is directly processed from the cytosol without the necessity for transport into the nucleus, it may be the better type of coding NA for GoF and LoF studies as long as no stable integration into the genome is intended. As discussed above, protein expression from pDNA requires mitosis to allow for pDNA transfer into the nucleus and the experimental concessions that come with it. Since mRNA is more directly turned into a protein without intracellular transport involved, it creates a promising and maybe even better alternative to pDNA.

### Spatially controlled delivery of siRNA for specific gene silencing (knock down)

#### Fluorescently labelled siRNA with random sequence (*siGLO Red™*)

**Fig. 6:**
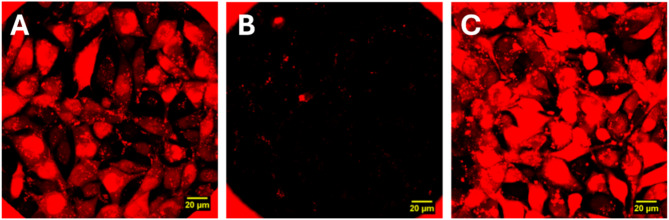
Delivery of *siGLO Red*™ into CHO-EGFP cells by means of *ISE*. (**A**) Electroporation of siRNA in a concentration of 2 µM by applying three sequential pulses with 4 V_rms_ at 40 kHz for 500 ms (8W4Eµ electrode array). Micrograph (**B**) represents the experimental outcome of cells incubated 2 µM *siGLO Red*^™^ similar to (A) but no electroporation pulse was applied. In (**C**) cells were chemically transfected with 33 nM *siGLO Red*^™^ using Lipofectamine^®^ RNAiMax. *siGLO Red*^™^ was localized using confocal scanning microscopy 75 min after electroporation (**A**, **B**) or 24 h after chemical transfection of CHO-EGFP cells (**C**).

Delivery of siRNA is another strategy to specifically reduce the concentration of a given protein and measure the associated *gain-* or *loss-of-function* in the cells of interest by impedance analysis. A fluorescently labelled, commercial siRNA (*siGLO Red™*), i.e. an oligonucleotide duplex with a randomized sequence designed as transfection tracer, was first delivered into confluent CHO-EGFP cells adherently grown on circular gold film electrodes (Applied BioPhysics Inc.; type 8W4Eµ, cf. Fig. 1) by means of *ISE*. It was the objective of these initial experiments to explore the general delivery of siRNA into adherent cells using the ECIS platform. The CHO-EGFP cells were engineered to express EGFP for later silencing of this reporter protein using a specific siRNA and conditions that were optimized for *siGLO Red*™. Cells were incubated with 2 µM *siGLO Red*™ in EBSS^++^ and subjected to three consecutive electroporation pulses using optimized pulse parameters (4 V_rms_ amplitude, 40 kHz AC frequency, 500 ms duration). As control, siRNA delivery was conducted in parallel by chemical transfection using 33 nM *siGLO Red*™ in combination with Lipofectamine^®^ RNAiMax. The red fluorescence emitted by the labeled siRNA served as marker to evaluate the transfection efficiency of both delivery approaches. Figure 6 illustrates the intracellular localization of *siGLO Red*™ in CHO-EGFP cells. Fluorescence microscopy images were acquired 75 min post-electroporation (**A**, **B**) and 24 h after chemical transfection (**C**). Efficient intracellular delivery of siRNA via *ISE* is proven by the red fluorescence localized throughout the cytoplasm of cells residing on the electrode (**A**) following pulse application with a preferential nuclear localization. In contrast, control cells that were incubated similarly with *siGlo Red*™ but did not receive any electroporation pulse showed no measurable red fluorescence (**B**). Despite significantly lower *siGlo Red*™ concentration in the extracellular fluid, chemical transfection yielded the highest efficiency of siRNA delivery as indicated by uniform staining of all cells across the field of view with no local confinement (**C**). The homogenous red emission in the periphery of the electrode (A, B) is due to the autofluorescence of the photopolymer that was used to delineate the circular electrodes by photolithography.

siRNAs are well-characterized for their high specificity and potency in gene silencing but their physicochemical properties (hydrophilicity, size and negative charge) prevent passive diffusion across the cell membrane^36^. Therefore, crossing the membrane barrier is crucial for siRNA applications in therapy or diagnostics. After *ISE*, we found *siGLO Red*™ molecules inside the cytoplasm with a preferential localization to the nucleus, which is also stated in the manufacturer’s application note^37^. However, the exact mechanism behind this preferred localization is not given. We can only assume, similar to aptamers, that electrostatic binding to histone proteins might be the primary reason for this preference. Chemical transfection of *siGLO Red*™ served as comparison to benchmark *ISE*-based delivery efficiency. Similar to the results observed for other nucleic acid species, chemical transfection provides superior loading with siRNA. However, notable differences in subcellular localization were observed microscopically. While chemical transfection resulted in a punctate fluorescence pattern, indicative of endosomal entrapment, *siGLO Red*™ introduced via *ISE* was predominantly found within the nuclei. These findings suggest that *ISE* enables more efficient cytosolic and nuclear delivery of siRNA compared to conventional lipid-mediated methods. Similar vesicular entrapment following Lipofectamine-based delivery was previously reported by *Breunig et al.*^38^ and corroborated by others^39,40^. These findings highlight the potential of *ISE* to rapidly deliver siRNA to its cytosolic sites of action avoiding a membrane-mediated uptake mechanism that results in membrane-confined siRNA molecules. Taken together, siRNA delivery was achieved using both, *ISE* and lipid-based chemical transfection, with both methods yielding good loading efficiencies.

### Silencing of EGFP expression (siRNA^EGFP^)

**Fig. 7:**
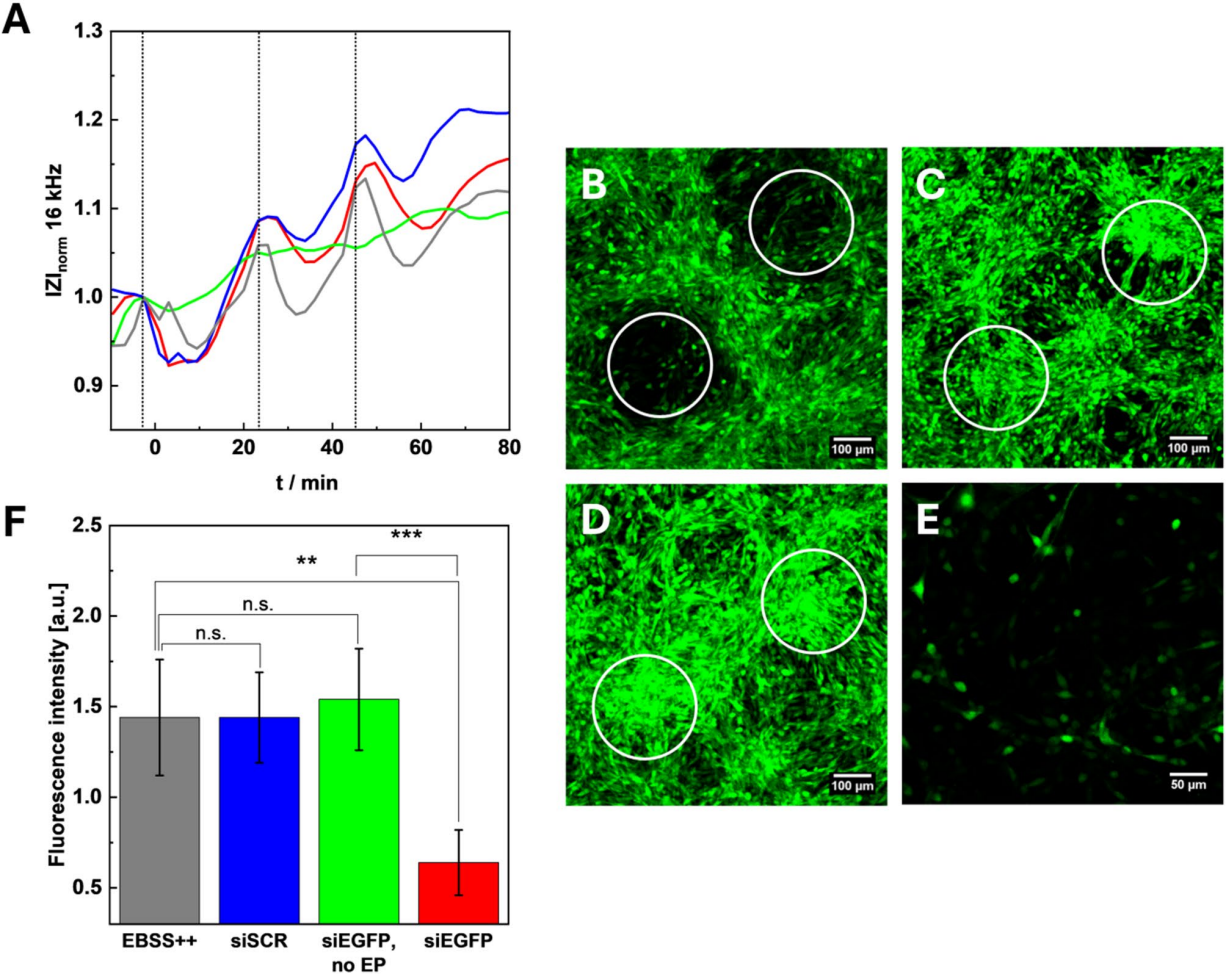
Delivery of siRNA^EGFP^ into confluent CHO-EGFP cells by means of *ISE*. (**A**) Impedance time courses of CHO-EGFP cells during the application of three consecutive electroporation pulses with 4 V_rms_ at 40 kHz for 500 ms (8W4Eµ electrode array). Cells were incubated with electroporation buffer (grey), with 2 µM siRNA^EGFP^ (red, green) or with 2 µM of siRNA^scr^ (blue). Three electroporation pulses were applied to all cell populations except for those represented by the green curve that did not receive any electroporation. Impedance was recorded at an AC frequency of 16 kHz and normalized to the last time point before the first electroporation pulse was applied. Times of electroporation are marked as dashed lines. (**B–D**) CLSM images of cell-covered electrodes 48 h after the electroporation pulses. (**B**) 2 µM siRNA^EGFP^, electroporation; (**C**) 2 µM siRNA^EGFP^, no electroporation; (**D**) 2 µM siRNA^scr^, electroporation. (**E**) Chemical transfection of CHO-EGFP cells using of 33 nM siRNA^EGFP^ together with Lipofectamine^®^ RNAiMax. (**F**) Quantification of the average fluorescence intensity within electrode areas (compare white circles in **B–D**) of cells receiving ISE in presence of buffer (grey), siRNA^EGFP^ (red) or siRNA^scr^ (blue). siRNA^EGFP^ loaded cells without receiving *ISE* are represented as green bar. Data are means ± SD (*n* = 4–8; n.s.: not significant *p* > 0.05, *: *p* < 0.05, **: *p* < 0.01, ***: *p* < 0.002).

Knockdown of EGFP in CHO cells, transfected to stably express EGFP (CHO-EGFP), was used as a functional proof for specific gene silencing in adherent mammalian cells when siRNA is delivered by *ISE*. Sense and antisense siRNA (22 nucleotides each) targeting the EGFP-mRNA (siRNA^EGFP^) was delivered to CHO-EGFP cells to reduce the copy number of EGFP proteins. Again, EGFP was used as a model system for assay development since the resulting protein concentration is readily measurable by fluorescence. CHO-EGFP cells were first grown to confluence on circular gold-film electrodes (Applied BioPhysics Inc., type: 8W4Eµ, cf. Fig. 1). For the delivery experiment, the cells were incubated with 2 µM siRNA^EGFP^ in EBSS^++^ before receiving three sequential electroporation pulses of 4 V_rms_ amplitude, 40 kHz AC frequency and 500 ms duration each. EGFP expression levels were assessed 72 h post-electroporation. Control conditions included (i) cells incubated with the same concentration of siRNA^EGFP^ but without pulse application and (ii) cells electroporated in presence of a commercial, non-targeting, scrambled control siRNA^scr^ (2 µM in EBSS^++^). Following electroporation, the assay medium was replaced with complete culture medium about 60 min after the final electroporation pulse. Chemical transfection of CHO-EGFP cells with 33 nM siRNA^EGFP^ and Lipofectamine^®^ RNAiMax served as positive control. Figure 7A shows the impedance profiles recorded before and after the individual electroporation pulses were applied to the cells.

For all experimental conditions (grey, blue and red curves), a rapid drop in impedance occurred after pulse application (dashed lines), followed by impedance recovery even beyond the pre-pulse impedance values within approximately 20 min. The green curve represents cells that were incubated with 2 µM siRNA^EGFP^ but did not receive an electroporation pulse. Even this curve showed a gradual increase in impedance with time indicating the unspecific nature of the impedance increase. This overlaying increase in impedance is most likely due to continuous cell growth and an increase of cell density on the electrode. Fluorescence micrographs, taken 48 h post-electroporation, are shown in Fig. 7B–D. Electrode borders are indicated by white circles. In cells that received *ISE* pulses in presence of siRNA^EGFP^ (**B**), reduced EGFP fluorescence was observed within and symmetrically around the gold-film electrodes (white circles) as compared to the surrounding area in the periphery of the electrode. As discussed before, we attribute this area of reduced gene expression that is bigger than the electrode as an indicator of cell proliferation and/or migration within 48 h after siRNA^EGFP^ delivery. The lower fluorescence intensities indicate localized gene silencing. This effect was neither observed in cells incubated with equal concentrations of siRNA^EGFP^ without receiving electroporation pulses (**C**) nor in cells that were electroporated in presence of a non-targeting, scrambled siRNA^scr^ (**D**). Cells residing on the electrodes in (C) and (D) show similar fluorescence levels as cells in the periphery of the electrodes. Fluorescence intensity recorded from cells residing on the electrodes (region of interest) was quantified using ImageJ. Data of the averaged pixel intensities from 4 to 8 independent experiments were pooled in the bar plot shown in Fig. 7F. Cells treated by *ISE* in absence of siRNA^EGFP^ (grey bar) or in presence of siRNA^scr^ (blue bar) showed similar average fluorescence intensities. Cells incubated with siRNA^EGFP^ without receiving electroporation pulses (green bar) led to EGFP fluorescence levels insignificantly higher than observed for the other controls. Significantly lower fluorescence levels were only found for those cells receiving *ISE* in presence of 2 µM siRNA^EGFP^ (red). This quantitative image analysis revealed 55% reduction in average pixel fluorescence intensity from cells within the electrode area exposed to siRNA^EGFP^, consistent with previous studies by *Fujimoto et al.*^41^. As a control for siRNA^EGFP^ delivery, CHO-EGFP cells were chemically transfected with 33 nM siRNA^EGFP^, resulting in a strong and uniform decrease in EGFP fluorescence across the entire well indicating a near-to-complete knockdown (**E**). However, in chemical transfection every cell of the population is treated the same and receives the siRNA^EGFP^ without any spatial patterning as in *ISE*. During the latter, delivery is confined to cells on the electrode which seems an exciting option for studies using OoC or MPS approaches providing experimental access for gene silencing in parts of the system while others are unaffected.

### Delivery of siRNAs inducing cell death (siRNA^cell−death^)

**Fig. 8:**
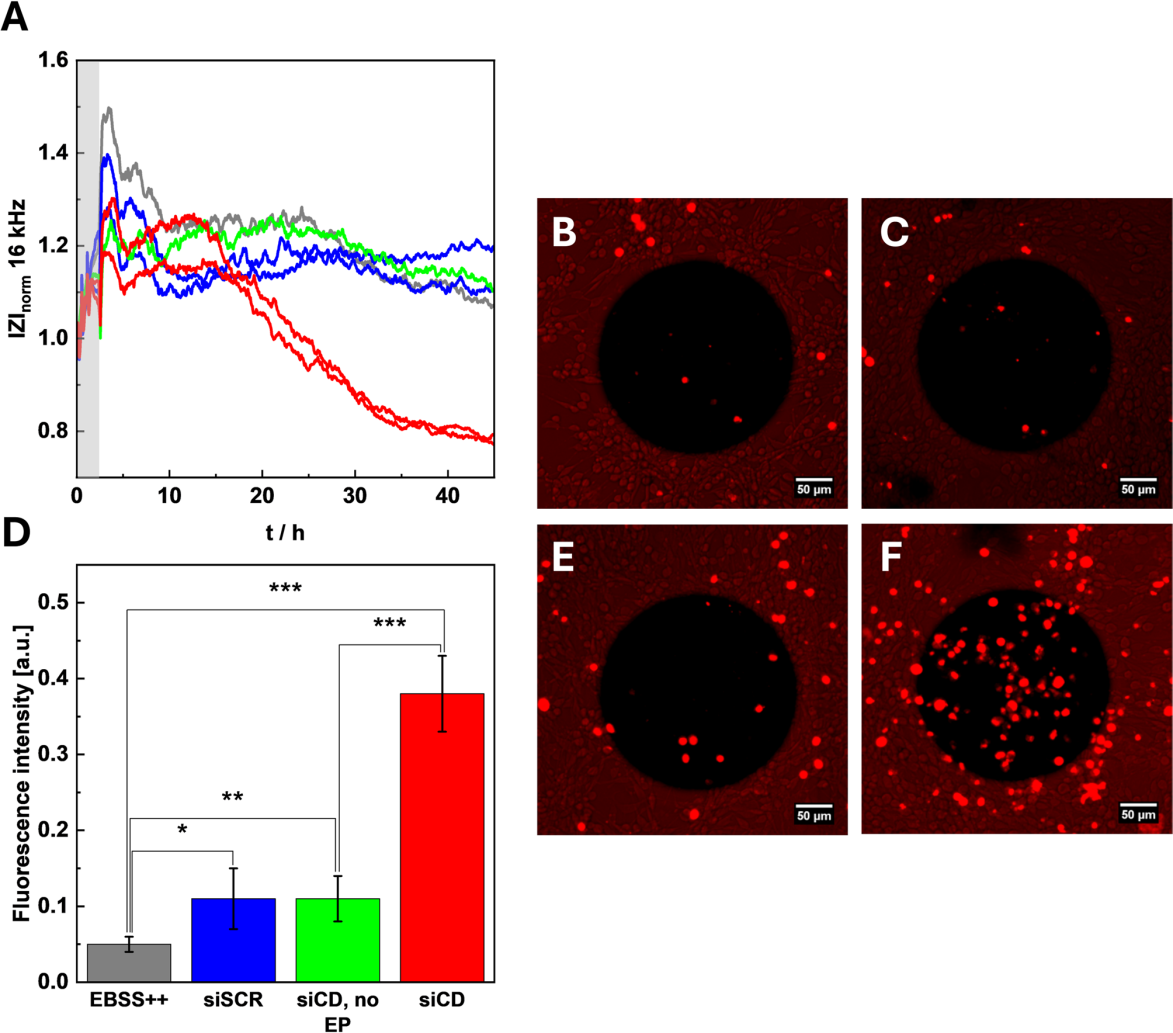
Delivery of siRNA^cell−death^ into CHO-EGFP cells by *ISE*. (**A**) Impedance time courses of adherent CHO-EGFP cell monolayers before and after a sequence of three electroporation pulses (4 V, 40 kHz, 500 ms; 8W4Eµ electrode array) when bathed in buffer (grey), supplemented with 2 µM siRNA^scr^ (blue) or 2 µM siRNA^cell−death^ (red). The green curve represents cells loaded with 2 µM siRNA^cell−death^ but without *ISE*. The time interval used to apply the electroporation pulses, exchange buffer and fill up the wells with medium after the final pulse is marked as grey box. Impedance was recorded at 16 kHz and normalized to the last data point before applying the first electroporation pulse. (**D**) Average fluorescence intensity of ethidium homodimer-stained cells within the electrode area (grey: buffer control, blue: 2 µM siRNA^scr^, electroporation; green: 2 µM siRNA^cell−death^, no electroporation; red: 2 µM siRNA^cell−death^, electroporation; mean ± SD, *n* = 4–8; *: *p* < 0.05, **: *p* < 0.01, ***: *p* < 0.002). (**B–F**) CLSM micrographs of CHO-EGFP cells on electrodes stained with EtHD 45 h after electroporation. (**B**) buffer control; (**C**): 2 µM siRNA^scr^, electroporation; (**E**) 2 µM siRNA^cell−death^, no electroporation; (**F**) 2 µM siRNA^cell−death^, electroporation.

In the sections above, we have demonstrated the successful delivery of siRNA by *ISE* and specific gene silencing on the example of EFGP. In this paragraph we have focused on the silencing of genes that are essential for cell survival so their silencing induces phenotypic changes that are sensitively measured by impedance readings. The sequence-specific, commercial pool of siRNAs^cell−death^ targets the transcripts of essential survival genes in both, mice and rats. Silencing of these genes triggers apoptosis, ultimately resulting in cell death^42^. To monitor the cellular response to siRNA^cell−death^ delivery, real-time impedance changes of gold-film electrodes covered with confluent monolayers of CHO-EGFP cells were recorded before and after *ISE* in presence of siRNA^cell−death^. Additionally, cells were stained by ethidium homodimer (EtHD) to quantify dead cells via fluorescence microscopy following siRNA^cell−death^ delivery. To control for off-target effects, a commercial scrambled siRNA^scr^ was included as a control. Cells were monitored 50 h post-electroporation via impedance measurements. Time courses are presented in Fig. 8A. The grey box denotes the experimental time of electroporation and subsequent buffer exchange. A transient increase in impedance was observed immediately after that period, likely due to cellular responses to liquid handling and medium addition. Impedance signals stabilized within 5 h. In contrast to control conditions (grey, blue and green curves), which remained largely stable throughout the experiment, cells electroporated in presence of 2 µM siRNA^cell−death^ (red curve) showed a decline in impedance starting around 20 h post-pulse gradually converging towards a new steady state. By the end of the observation period, normalized impedance values had decreased to 0.8, suggesting morphological changes consistent with cell rounding as a consequence of the onset of apoptosis.

To assess cell viability, cells were stained with *ethidium homodimer* and studied by CLSM. Ethidium homodimer is a fluorescent indicator for dead cells with permeabilized membranes similar to *propidium iodide* used in a preceding set of experiments. Representative micrographs are shown in Figs. 8B, C, E, F. Control conditions (**B**, **C**, **E**) displayed a low number of permeabilized cells (indicated by bright red nuclear fluorescence), presumably reflecting the normal percentage of dead cells in any cell population in vitro. When cells were exposed to 2 µM siRNA^cell−death^ during *ISE* (**F**), a significantly larger number of permeabilized and dead cells is clustered around the active electrode used for pulse application and siRNA^cell−death^ delivery. Dead cells were not only found on the electrodes but also in their periphery which is in line with results obtained after siRNA^EGFP^ electroporation. Quantification of fluorescence intensities within electrode areas from 4 to 8 micrographs per condition was performed using ImageJ and is summarized in Fig. 8D. The controls (grey, blue and green bars) showed relatively low fluorescence intensities compared to the average intensity of micrographs recorded for cells that were electroporated in presence of siRNA^cell−death^. Electroporation in presence of siRNA^scr^ (blue bar) and incubation with siRNA^cell−death^ without *ISE* (green bar) also led to slightly elevated levels of cell damage. This may suggest some nonspecific, cytotoxic effects of siRNA^scr^ and possible endocytotic entry of siRNA^cell−death^ in cells not receiving *ISE*. At the same time, this phenomenon could be the result of stochastic distribution of dead cells within the electrode area or beyond. These experiments demonstrate also on the level of siRNA that specific intracellular manipulation of adherent cells in concert with time-resolved impedance measurements opens up a new experimental strategy to study phenotypic *loss-* or *gain-of-function* in mammalian cells.

## Conclusion

This study highlights the broad applicability of *in situ electroporation* (*ISE*) as a versatile and efficient method for the intracellular delivery of diverse types of coding nucleic acids, including aptamers, plasmid DNA, mRNA, and siRNA into adherent mammalian cells. Even though the pure transfection efficiency is often times higher with conventional chemical transfection, *ISE* outperforms the former by enabling direct cytosolic access without the need for endosomal escape. Moreover, since *ISE* does not rely on chemical additives with often unknown impacts on cell physiology and allows for cell recovery within less than an hour, intracellular delivery is conducted less invasive. Spatial precision is another key advantage of *ISE*, allowing for localized delivery to defined sub-populations of cells within intact cell monolayers. This particular feature may pave the way to rather new assays addressing phenotypic differences in neighboring cells with precisely-defined engineering of their proteomes. Since *ISE* is readily compatible with non-invasive impedance monitoring using technologies such as ECIS, changes in cell function may get disclosed with temporal resolution for observation times from minutes to hours or days. Importantly, pulse parameters can be precisely tuned and adapted to the specific requirements of different cell types, underscoring the method’s flexibility and broad utility across different experimental settings. Lastly, *ISE*-ECIS is readily integrated even in microfluidic cell culture vessels (e.g. organ-on-chip) or high throughput multi-well arrays so that application to more complex in vitro models or screening campaigns seems straightforward.

## Supplementary Information

Below is the link to the electronic supplementary material.

Supplementary Material 1

## Data Availability

Research data will be made available to the community upon request.
