# Supplementary material for: Localized delivery of coding nucleic acids into adherent cells by in situ electroporation: integrated impedance-based monitoring allows for loss-of-function or gain-of-function screening
Source: Sci Rep. 2026 Jul 29;16:23544. doi: 10.1038/s41598-026-63882-5 (PMC13421483; doi:10.1038/s41598-026-63882-5)
Supplement: Supplementary file 1 — Supplementary Material 1 [file 41598_2026_63882_MOESM1_ESM.docx]

**Localized Delivery of Coding Nucleic Acids into Adherent Cells by in situ Electroporation: Integrated Impedance-based monitoring allows for loss-of-function or gain-of-function screening**

Anne-Kathrin Grimm^1*^, Sonja Balk^1*^, Achim Göpferich^2^, Miriam Breunig^2^, Simone Aubele^3^, Anne Zemella^3^, Joachim Wegener^1,4^

Supplementary Information

**Intracellular localization of aptamers after *in situ* electroporation**

After intracellular delivery of the fluorescence-labeled aptamer probe into confluent layers of NRK cells by *in situ* electroporation (three pulses), cells were stained with the lysosomal marker LysoTracker® Blue DND-22 to assess the intracellular localization of the aptamer. This membrane-permeable probe selectively accumulates in acidic intracellular compartments, primarily lysosomes, in living cells. Cells were incubated with 1 µM dye in culture medium for 1 hour in the dark. Excess extracellular dye was removed by washing the cells twice with PBS.

Fluorescence micrographs revealed predominant nuclear localization of the aptamer in a punctate pattern, which appeared brighter than the surrounding nucleoplasm (cf. E). Co-localization analysis of the lysosomal tracker signal (blue) and aptamer signal (green) showed no detectable overlap, indicating that the aptamer was not retained in lysosomes but accumulated mainly in the nucleus.


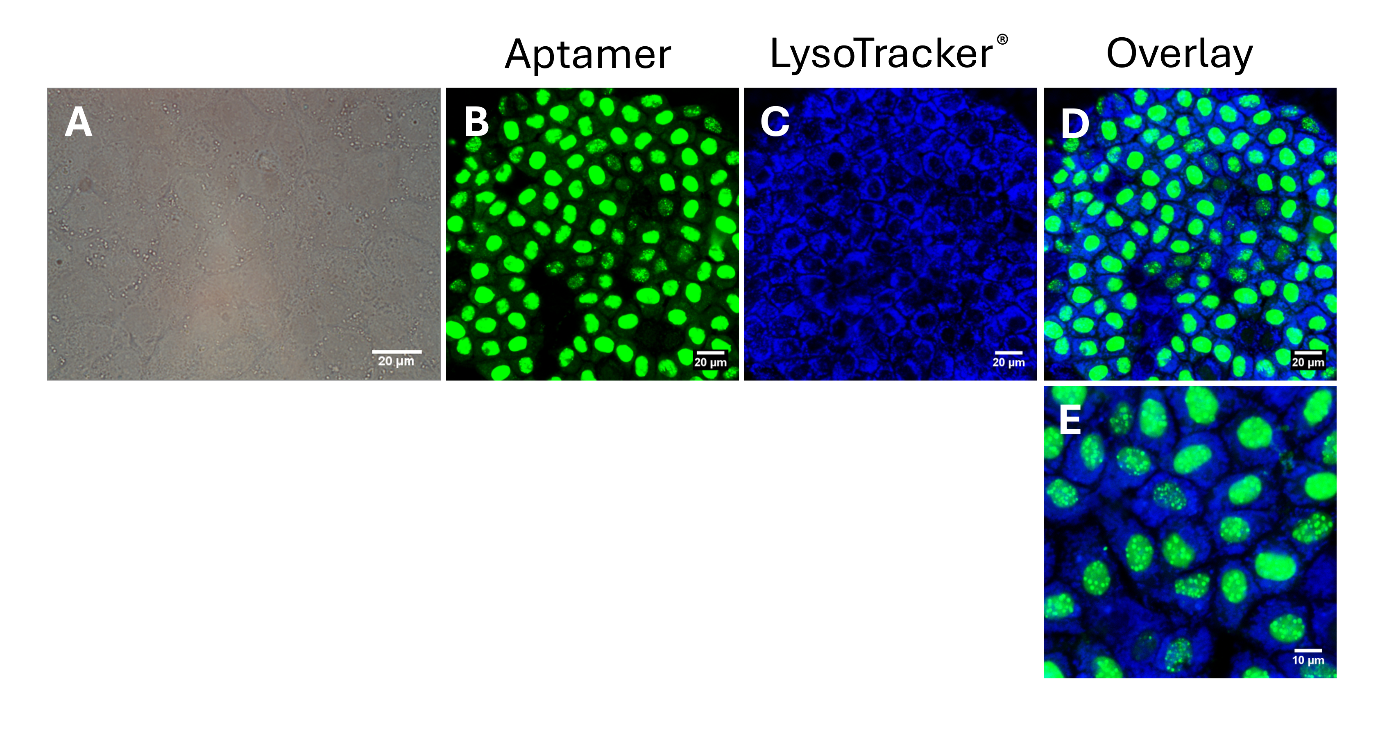


**Supplementary Information 1**: (**A**) Phase-contrast image and (**B-E**) confocal fluorescence micrographs of NRK cells following in situ electroporation with 15 µM aptamer (green). After electroporation, cells were incubated for 1 h with the lysosomal marker LysoTracker® Blue DND-22 (blue) prior to microscopic analysis.
